# The Structure of a Novel Thermophilic Esterase from the Planctomycetes Species, *Thermogutta terrifontis* Reveals an Open Active Site Due to a Minimal ‘Cap’ Domain

**DOI:** 10.3389/fmicb.2015.01294

**Published:** 2015-11-23

**Authors:** Christopher Sayer, Zalan Szabo, Michail N. Isupov, Colin Ingham, Jennifer A. Littlechild

**Affiliations:** ^1^The Henry Wellcome Building for Biocatalysis, Biosciences, College of Life and Environmental Sciences, University of ExeterExeter, UK; ^2^Microdish BVUtrecht, Netherlands

**Keywords:** Planctomycetes, thermophilic enzymes, carboxyl esterase, X-ray structure, biocatalysis

## Abstract

A carboxyl esterase (TtEst2) has been identified in a novel thermophilic bacterium, *Thermogutta terrifontis* from the phylum Planctomycetes and has been cloned and over-expressed in *Escherichia coli*. The enzyme has been characterized biochemically and shown to have activity toward small *p*-nitrophenyl (*p*NP) carboxylic esters with optimal activity for *p*NP-acetate. The enzyme shows moderate thermostability retaining 75% activity after incubation for 30 min at 70°C. The crystal structures have been determined for the native TtEst2 and its complexes with the carboxylic acid products propionate, butyrate, and valerate. TtEst2 differs from most enzymes of the α/β-hydrolase family 3 as it lacks the majority of the ‘cap’ domain and its active site cavity is exposed to the solvent. The bound ligands have allowed the identification of the carboxyl pocket in the enzyme active site. Comparison of TtEst2 with structurally related enzymes has given insight into how differences in their substrate preference can be rationalized based upon the properties of their active site pockets.

## Introduction

The esterase enzymes carry out the hydrolysis of esters into an acid and an alcohol. These ubiquitous enzymes are of considerable physiological significance being widely distributed in bacteria, archaea, and eukaryotes (Montella et al., 2012). The esterases are widely used as stereoselective catalysts for the synthesis of optically pure molecules for the pharmaceutical and agrochemical industries (Faber, 1997). Their extensive application in industry is due to their versatility, robustness, stereoselectivity and their ability to promote synthetic reactions in organic solvents (Bornscheuer, 2002). Esterases are commonly used in the resolution of chiral carboxylic acids and primary, secondary and tertiary alcohols. Examples include the release of ferulic acid from plant cell wall polysaccharides such as pectin or xylan (de Vries and Visser, 1999) and the catabolism of benzyl esters with the release of acid and benzyl alcohols (Jones et al., 1999). These products are subsequently oxidized to benzoates by dehydrogenases and further metabolized in the β-ketoadipate pathway. The carboxyl esterase NP is used for the production of the non-steriodal drug naproxen (Quax and Broekhuizen, 1994). The racemic naproxen methylester is hydrolysed to the *(S)*-acid which is separated from the *(R)*-methylester to yield *(S)*-naproxen with a 99% ee and a yield of 95%.

Carboxyl esterases (EC 3.1.1.1) and lipases (EC 3.1.1.3) are enzymes that catalyze the cleavage of an ester bond between a carboxylic acid and an alcohol group. The carboxyl esterases utilize relatively small water soluble substrates, whilst lipases catalyze a related reaction on larger water insoluble long-chain fatty acids which involve the process of interfacial activation (Schrag and Cygler, 1997).

The first crystal structure of an esterase was that of acetyl-choline esterase from *Torpedo californica* (Sussman et al., 1991). Most of the carboxyl esterases and the lipases belong to the α/β hydrolase fold family (Ollis et al., 1992; Heikinheimo et al., 1999). The α/β hydrolase fold family forms one of the largest groups of structurally related proteins with a wide diversity of catalytic and non-catalytic functions (Carr and Ollis, 2009). The fold consists of an eight stranded β sheet with the second strand antiparallel to the others with the strand order 1,2,4,3,5,6,7,8 and a catalytic triad of Ser-His-Asp(Glu). The α/β hydrolase fold shows a large structural versatility in which sequences with low levels of amino acid similarity can adopt the same structural core. To obtain the vast variety of α/β hydrolase enzymes different additional domains are added to the core structure that are responsible for the differences in enzymatic activities within the one overall fold (Marchot and Chatonnet, 2012). A distinguishing feature of lipases is that they have a movable lid which is involved in the process of ‘interfacial activation’ (Brzozowski et al., 1991). Amongst the many esterase enzymes reported the variations in the ‘cap’ domain are responsible for the differences in substrate specificities (Holmquist, 2000). There are a few examples of some enzymes which lack parts or most of the ‘cap’ domain such as the *Streptomyces exfoliates* lipase (Wei et al., 1998) and the esterase from *Lactobacillus plantarum* (Benavente et al., 2013). There are some members of the α/β hydrolase family with smaller β-sheets such as the *Penicillium purpurogenum* acetylxylan esterase (AEXII) which contains a five stranded β sheet and also lacks a full ‘cap’ domain (Ghosh et al., 1999). The carboxyl esterase activity has also been reported for some enzymes which have different folds (Wei et al., 1995), including an α/β/α hydrolase fold (Upton and Buckley, 1995), β-lactamase fold (Wagner et al., 2002; Isupov et al., 2004) and as a side activity for the carbonic anhydrase enzymes (Höst et al., 2006; James et al., 2014).

The proposed mechanism of catalysis for carboxyl esterases resembles the serine protease mechanism described by Blow et al. (1969). First the substrate carbonyl oxygen adjacent to the scissile bond binds to the oxyanion hole, which is usually formed by two main chain nitrogen atoms. A tetrahedral intermediate is then formed as a result of the nucleophilic attack by the catalytic serine hydroxyl on the carbonyl carbon. Release of the alcohol product is followed by an attack of an acyl-enzyme complex by a water molecule. Collapse of a resulting tetrahedral intermediate leads to the release of the carboxyl product and the free enzyme. The catalytic serine residue in the α/β hydrolase fold enzymes is usually located in a tight nucleophilic elbow with the consensus sequence Gly-X-Ser-X-Gly (Nardini and Dijkstra, 1999) however, both glycines are not conserved in the *Alcaligenes* esterase (Bourne et al., 2000).

The importance of hydrolytic enzymes for industrial applications is a driving force to search for new enzymes from extremophilic microorganisms. These enzymes are more robust than their mesophilic counterparts making them more stable in a variety of commercial processes (Littlechild et al., 2007). Features of enzymes from thermophiles, which are stable at high temperatures, stable in organic solvents, have broad substrate specificity and high regio- and stereo-selectivity, make these enzymes attractive biocatalysts (Littlechild et al., 2004). Many thermophilic esterases have been characterized, including two from *Archaeoglobus fulgidus* (De Simone et al., 2001; Kim et al., 2008) and others from *Rhizomucor miehei* (Yang et al., 2015), *Sulfolobus tokadaii* (Angkawidjaja et al., 2012), *Thermobifida fusca* (Billig et al., 2010), *Alicyclobacillus acidcaldarius* (Mandrich et al., 2008), *Geobacillus stearothermophilus* (Liu et al., 2004, 2007) and *Pyrobaculum calidifontis* (Palm et al., 2011), amongst others.

The enzyme described within this paper was an output from the EU funded ‘Hotzyme’ project which aimed to discover new hydrolytic enzymes from thermophilic genomes and metagenomes with potential industrial applications. Many novel hydrolase enzymes have been identified and characterized during this project including a lactonase from *Vulcanisaeta moutnovskia* (Kallnik et al., 2014), two epoxide hydrolases from thermophilic metagenomes (Ferrandi et al., 2015), and an esterase from *Thermogutta terrifontis* (TtEst), which demonstrated activity to short chain esters (C1-3; Sayer et al., 2015). The moderately thermophilic *T. terrifontis* has been isolated from a terrestrial hot spring on Kunashir Island, Russia (Slobodkina et al., 2014). It is the first thermophilic member of the phylum Planctomycetes. This organism has an optimum temperature for growth of 55–60°C.

This paper describes the identification, cloning, biochemical and structural characterization of a second esterase from *T. terrifontis* (TtEst2). This enzyme which belongs to the Pfam α/β hydrolase 3 family was cloned and over-expressed in *Escherichia coli*. The purified TtEst2 enzyme has been crystallized and the structures have been determined of both the native enzyme and its complexes with three different reaction products. This information has provided an insight into the substrate specificity of this novel thermophilic carboxyl esterase with respect to its exposed active site and minimal ‘cap’ domain when compared to other members of the α/β hydrolase 3 family.

## Materials and Methods

### Cloning and Over-expression

Potential hydrolase sequences were identified in the *T. terrifontis* genome using the ANASTASIA galaxy pipeline (Pilalis et al., 2012; Ladoukakis et al., 2014).

One gene that encoded a secreted protein with an α/β-hydrolase 3 domain (Pfam07859; Finn et al., 2014) was cloned for heterologous expression in *E. coli.* The signal peptide cleavage site was predicted with SignalP 4.0 (Petersen et al., 2011) and the sequence encoding the gene without the signal peptide was amplified by polymerase chain reaction (PCR), using the primers O-040 (GGTTGGGAATTGCAAGCCGAGGTGGGGCGGCTTC) and O-041 (GGAGATGGGAAGTCACTACGGTTGAGACTCTCCCTTG). The PCR product was cloned by ligation independent cloning using an aLICator kit into the pLATE52 vector (Thermo Scientific) which adds an amino-terminal hexa-histidine tag followed by a WELQ protease cleavage site. The cloned sequence was verified by Sanger sequencing (GATC Biotech, Konstanz, Germany).

The *E. coli* BL21 (DE3) cells harboring the *TtEst2* plasmid were cultured overnight in LB medium (100 mls). The overnight culture was used to inoculate 1 L of LB media and incubated at 37°C with shaking at 250 rpm until an OD_600_ of 0.6 was reached prior to being induced with the addition of 0.5 mM IPTG. The cultures were left shaking at 37°C for ∼4 h. The cells were then harvested at 4700 g at 4°C for 20 min. The pellet was then re-suspended in 25 mM Tris-HCl pH 8.0, 0.5 M NaCl, 20 mM imidazole. The cells were disrupted by sonication at 10 microns (Soniprep 150, MSE, London, UK) on ice for 4 min and the cell debris removed by centrifugation at 20,000*g* at 4°C for 30 min. The clarified cell lysate was then heat-treated at 65°C for 30 min before being centrifuged at 20,000*g* at 4°C for 30 min to remove any denatured proteins.

The TtEst2 was purified on a 1 ml His-Trap FF crude column (GE Healthcare, Little Chalfont, UK) using an elution gradient from 20 to 500 mM imidazole in 20 mM Tris-HCl pH 8.0, 0.5 M NaCl. The enzyme was then applied to a calibrated Superdex 200 HiLoad 16/60 gel filtration (GF) column (GE Healthcare, Little Chalfont, UK) and was eluted with 1 column volume in a buffer of 25 mM Tris-HCl pH 7.5, 100 mM NaCl at 1.0ml/min.

### Activity Assays

Activity for the hydrolysis of an ester was determined at 25°C measuring *p*-nitrophenyl (*p*NP) production from its carboxylic esters *p*NP-acetate, *p*NP-propionate, *p*NP-butyrate, and *p*NP-valerate, *p*NP-hexanoate and *p*NP-octanoate (Armstrong et al., 1966). Reactions were carried out in a total volume of 100 μl containing a final concentration of 20 mM HEPES buffer, 100 mM NaCl pH 7.5, 10 μg/ml^-1^ enzyme, 1 mM substrate and the change in A_410_ was recorded.

The thermal stability of TtEst2 was tested by incubating the enzyme at a concentration of 1 mg/ml in 20 mM HEPES, 0.5 M NaCl pH 8.0 at 50, 60, 70, 80, 90, and 100°C for 30 min. Enzyme aliquots (100 μl) were withdrawn at appropriate times and cooled on ice before the residual activity was measured using the method described above.

### Crystallization

The TtEst2 was concentrated to ∼15 mg/ml using a 10 kDa membrane Vivaspin (Vivaproducts, Littleton, MA, USA) and microbatch crystallization trials were set up using an Oryx 6 crystallization robot (Douglas Instruments, Hungerford, UK) using the The Stura Footprint Screen^TM^. The droplet contained a 50:50 ratio of protein solution to screen and was covered with Al’s oil (50:50 mix of silicon and paraffin oils) before being stored at 20°C and was regularly checked for growth of crystals using a light microscope.

Crystals appeared within 1 week and the native crystals were grown from 50 mM Na HEPES pH 7.5 and 15% PEG600. Some crystals were frozen directly from the droplet and the rest were frozen using a cryoprotectant consisting of 50 mM Na HEPES pH 7.5, 100 mM NaCl and 30% PEG400. To obtain ligand complexes crystals were soaked for 30–300 s in a cryoprotectant of 50 mM potassium hydrogen phthalate buffer pH 4.5, 100 mM NaCl and 30% PEG400 containing either 25 mM propionate, butyrate or *p*NP-valerate.

### X-ray Data Collection and Structure Solution

Data were collected on beamline I04-1 at the Diamond Synchrotron light source (Didcot, UK) at 100 K in a stream of gaseous nitrogen using a Pilatus detector (Dectris Ltd, Baden, Switzerland). Native data were processed using XDS (Kabsch, 2010), the data from ligand complexes were processed with DIALS (Gildea et al., 2014). Data were scaled using AIMLESS (Evans and Murshudov, 2013) in the Xia2 pipeline (Winter et al., 2013). All further data and model manipulation was carried out using the CCP4 suite of programs (Winn et al., 2011).

The phases for the native structure were determined using the molecular replacement (MR) method implemented in MOLREP (Vagin and Teplyakov, 2010) using the assembly containing superimposed monomers of *A. acidocaldarius* esterase (AaEst2; PDB 1EVQ; De Simone et al., 2000) and metagenomic thermophilic carboxylesterase (EstE1; PDB 2C7B; Byun et al., 2007) which both share 29% sequence identity with TtEst2. The two models were first subjected to the MOLREP model modification based on the sequence alignment (Lebedev et al., 2008) and then were superimposed in COOT (Emsley et al., 2010). The rotation function was calculated at 3 Å resolution with an integration radius of 28 Å but failed to give any significant solutions. A subsequent translational MR search at 4.2 Å with the first 40 peaks of RF produced what appeared to be a promising solution, with a first translation peak for the first peak of RF with a score of 13.3. The background score for this rotation peak was below 11 and the translation function score did not exceed 12.3 for the remaining rotation peaks. The resulting solution could not be refined for any of the models either using the ARP/wARP procedure (Langer et al., 2013) or Refmac5 (Murshudov et al., 2011). Therefore the MR solution was subjected to 30 cycles of SHELXE phase extension procedure (Thorn and Sheldrick, 2013), with the correct model built at cycle 11. The resulting model, which contained 259 alanine residues, had a correlation for the partial structure against the native data of 50.26% (a value of over 30% usually indicates a correct structure), with only 19% of the starting Cα atoms of the MR model being within 1 Å of their positions in the SHELXE model.

The resulting SHELXE structure was subjected to the ARP/wARP procedure, prior to manual model building in COOT followed by refinement with REFMAC5. The dictionary definitions for the ligands were created using JLIGAND (Lebedev et al., 2012). The statistics of the data processing and parameters of the final refined models are given in **Table 1**. The quality of the model was checked using the program PROCHECK (Laskowski et al., 1993). Images were created using the molecular graphics programs PyMol (DeLano, 2002) and ccp4mg (McNicholas et al., 2011). To find the best data set amongst several which were collected for each complex the CCP4 program DIMPLE (Winn et al., 2011) was used.

**Table 1:** Summary of the data processing and refinement statistics.

Crystal	Native	Propionate	Butyrate	Valerate
Beamline	I04-1	I04-1	I04-1	I04-1
Wavelength (Å)	0.91741	0.91731	0.91731	0.91731
Space croup	P2_1_2_1_2_1_	P2_1_2_1_2_1_	P2_1_2_1_2_1_	P2_1_2_1_2_1_
Unit cell a,b,c (Å) α,β,γ (°)	61.6, 71.1, 75.8, 90, 90, 90	62.0, 70.9, 75.9, 90, 90, 90	61.8, 70.8, 75.6, 90, 90, 90	62.0, 70.9, 75.9, 90, 90, 90
Resolution range (Å)	47.80–1.58 (1.62–1.58)^a^	51.80–1.71 (1.80–1.71)	70.60–1.79 (1.90–1.79)	51.80–1.79 (1.90–1.79)
Multiplicity	6.7 (7.0)	6.2 (5.8)	6.3 (6.4)	6.1 (6.2)
Unique reflections	46778	36869	31702	32257
Completeness (%)	99.9 (99.9)	100.0 (100.0)	100.0 (100.0)	100.0 (100.0)
R_sym_^b^ (%)	7.1 (87.4)	6.6 (112.8)	11.0 (122.0)	8.9 (110.2)
I/σ (*I*)	13.3 (2.0)	12.9 (1.5)	7.6 (2.0)	9.8 (1.6)
Wilson B factor (Å^2^)	29.1	31.1	31.8	30.5
Refined residues	1–172, 176–283	6–171, 177–282	5–282	1–171, 177–282
Refined water molecules	208	228	193	214
Refined ligand^∗^ molecules	-	1	1	1
R_cryst_^c^ %	15.01	16.81	17.55	18.46
R_free_ % (5% of total data)	17.49	20.22	22.08	24.10
R.m.s.d. bond lengths (Å)	0.010 (0.019)^d^	0.008 (0.019)	0.010 (0.019)	0.010 (0.019)
R.m.s.d. bond angles (^o^)	1.49 (1.98)	1.35 (1.98)	1.46 (1.97)	1.46 (1.98)
Occupancy of ligand	-	1.0	1.0	1.0
**Average B factor (Å^2^)**				
Protein	27.8	28.1	30.9	28.9
Solvent	42.2	41.7	40.9	40.4
Ligands	-	33.6	35.0	29.8
**Ramachandran plot analysis, residues in (%)**				
Most favored regions	90.1	89.0	89.3	89.2
Additional allowed regions	8.6	10.1	9.8	10
Generously allowed regions	0.9	0.4	0.4	0.4
Disallowed regions	0.4	0.4	0.4	0.4

*^a^Values in parentheses are given for the outer resolution shell.*

*^b^R_*sym*_ = Σ_*h*_ Σ_*J*_| <I_*h*_>-I_*J*_ (h)| /Σ_*h*_Σ_*J*_I_*J*_ (*h*), where I(h) is the intensity of reflection h. Σ_*h*_ is the sum over all reflections and Σ_*J*_ is the sum over J measurements of the reflection.*

*^c^*R*_*cryst*_ = Σ|| Fo| -| Fc|| /Σ| Fo|.*

*^d^Target values are given in brackets.*

*^∗^Only molecules within the active site cavity are considered ligands. Wilson B-factor was estimated by SFCHECK (Vaguine et al., 1999). Ramachandran plot analysis was performed by PROCHECK (Laskowski et al., 1993).*

## Results and Discussion

### Biochemical Characterisation

**FIGURE 1:**
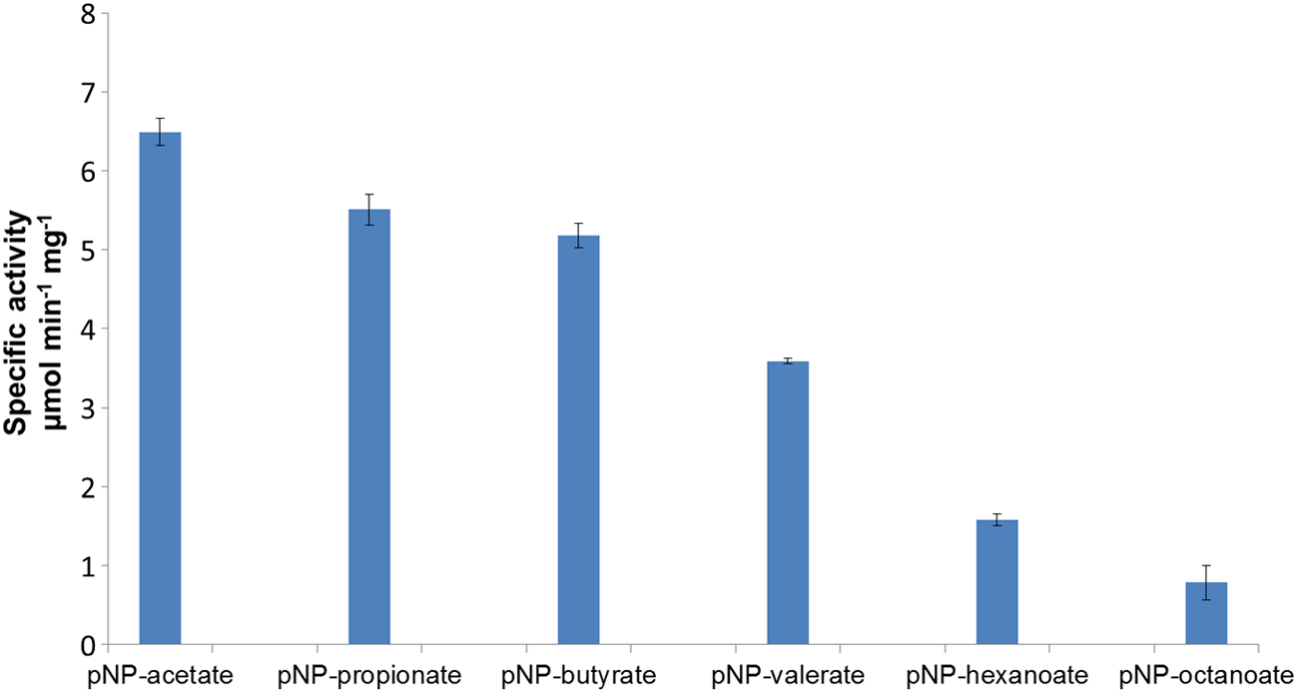
**The specific activity of the native TtEst2 toward *p*NP-esters with varying carbon chain lengths.** The enzyme activity was measured by monitoring *p*NP production (Armstrong et al., 1966).

The TtEst2 enzyme was successfully over-expressed in the *E. coli* BL21 (DE3) strain and purified using a nickel affinity column and gel filtration chromatography. The protein eluted as a monomer from a calibrated size exclusion column. The esterase activity was tested using short chain *p*NP esters as substrates (**Figure 1**). The enzyme was most active toward *p*NP-acetate (5.9 μM sec^-1^mg^-1^) with decreasing activity toward increasingly larger substrates with limited activity to *p*NP-octanoate. The thermostability of the enzyme was tested by incubation at increasing temperatures, cooling, and measuring the residual remaining activity. This procedure showed the enzyme had moderate thermostability retaining 90% of its activity after incubation for 30 min at 60°C and 75% of its activity at 70°C. No activity was observed after incubation at 80°C or above suggesting that TtEst2 is less thermostable than the previously characterized TtEst (Sayer et al., 2015).

### Structure of TtEst2

#### Quality of the Models

The TtEst2 enzyme crystallized in the space group P2_1_2_1_2_1_ with unit cell parameters *a* = 61.6, *b* = 71.1, *c* = 75.8 Å, α = β = γ = 90°. The native structure was refined with isotropic B-factors at a resolution of 1.6 Å with a final *R*_free_ of 17.5% (**Table 1**). The crystals were soaked in the presence of propionate, butyrate and *p*NP-valerate with the data of ligand complexes collected to a resolution of 1.8 Å or better with the resulting structures refined to an *R*_free_ of 24% or better. The asymmetric unit contains a single monomer giving a solvent content of 53% (*V*m = 2.64). As we have previously observed for the TtEst enzyme (Sayer et al., 2015), the structures appear to differ when the experiment is carried out at pH 4.5 or pH 7.0, in particular with regards to the occupancies of different rotamers of the protein side chains. Also the disordered loops were modeled differently between the native structure of TtEst2 and structures of the low pH ligand complexes. Residues which could not be modeled into electron density in the four different structures are listed in **Table 1**. Many residues had multiple conformations of side chains in all structures. Residues Pro40 and Ala41 were modeled in a single conformation in the butyrate complex structure and with two conformations of the main chain in the native structure and the other complexes. One of conformers of the split main chain Ala41 and the active site Asn161 are in the generously allowed region of the Ramachandran plot (Ramakrishan and Ramachandran, 1965). The active site Ser126 is in the disallowed region of the Ramachandran plot, as observed in the structures of most other α/β hydrolase fold enzymes (Ollis et al., 1992; Line et al., 2004). Pro9 is in a *cis* conformation in all of the TtEst2 structures.

#### Overall Fold and Closest Homologs

**FIGURE 2:**
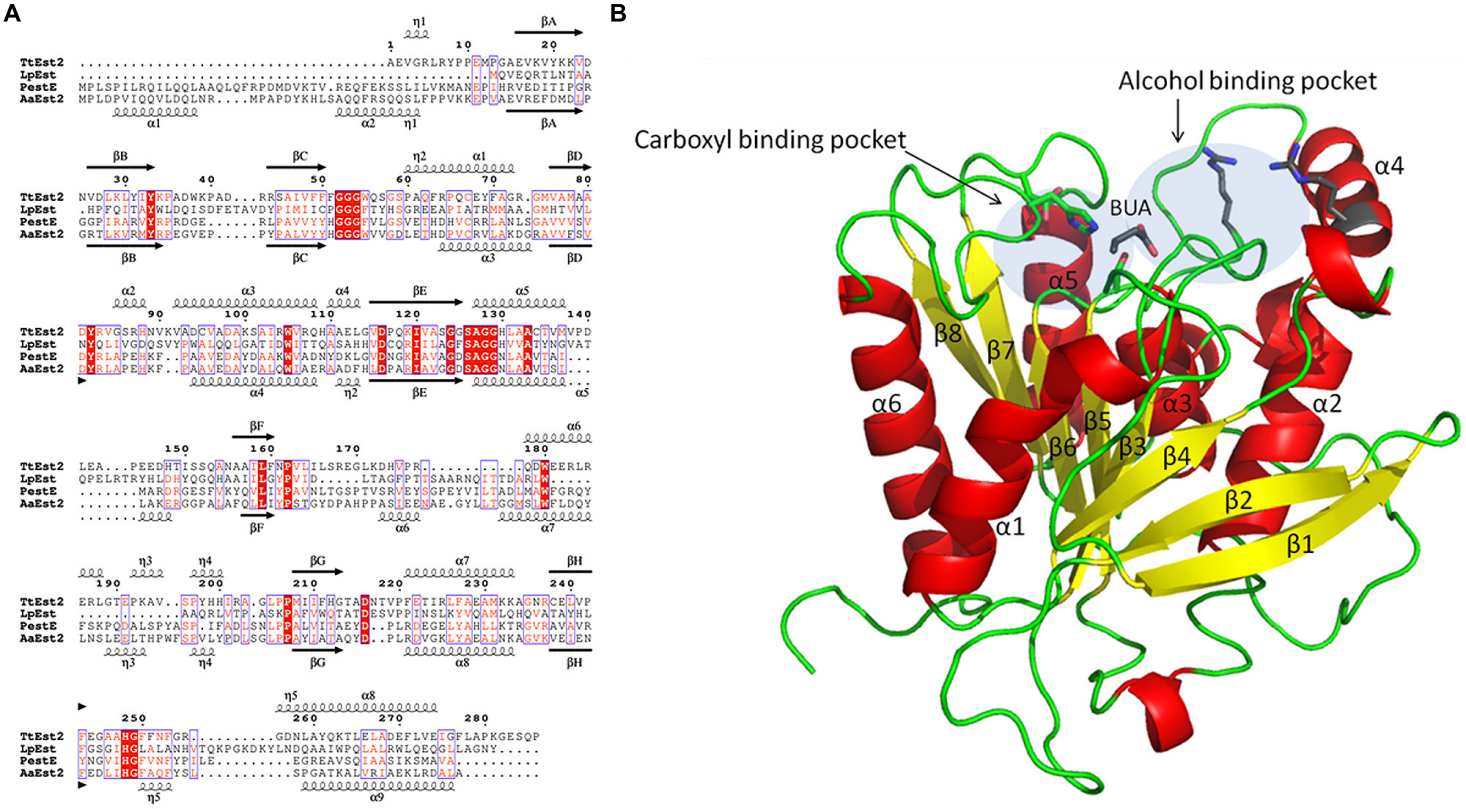
**(A)** Amino acid sequence alignment of TtEst2, LpEst, PestE, and AaEst2 shown with the secondary structure elements indicated above and below the sequence for TtEst2 and AaEst2 enzymes. Spirals and arrows represent α-helices, η-3_10_ helices and β-strands, respectively. The secondary structure assignments were carried out and the figure was produced using ESPript (Robert and Gouet, 2014). Conserved residues are shown in red boxes, and residues with similar properties are in blue boxes. **(B)** Folding of the TtEst2 monomer presented as a cartoon diagram. The residues of the catalytic triad are shown as stick model with carbon atoms in green, the butyrate ligand (BUA) which maps the carboxyl binding pocket is shown with carbon atoms in gray, arginine residues at the far end of alcohol pocket are shown with carbon atoms in gray. The two transparent boxes outline the carboxyl and the alcohol binding pockets. **Figures 2B, 3**, and **4** were prepared using PyMol (DeLano, 2002).

The TtEst2 is a monomer in the crystal, consistent with the results obtained from size exclusion chromatography. The TtEst2 monomer (**Figures 2A,B**) is made up of a α/β hydrolase fold core domain (Holmquist, 2000). Five long helices surround an eight stranded β-sheet with connectivity 1,2,-1x,2x,1x,1x,1x and direction + - + + + + + + (Richardson, 1981). The active site is located at the top of the β sheet and consists of the catalytic triad of Ser126, His 248 and Asp216. The catalytic serine is located in a tight nucleophilic elbow at the end of strand β5 which contains the conserved α/β-hydrolase signature motif.

TtEst2 belongs to the α/β-hydrolase family 3 in the Pfam classification (Finn et al., 2014). While multiple enzymatic activities have been reported for the α/β-hydrolase enzymes (Marchot and Chatonnet, 2012) family 3 appears to contain mostly carboxyl esterases. A BLAST (Altschul et al., 1990) search revealed a putative esterase from another Planctomycetes species *Blastopirellula marina* as a closest sequence homolog of TtEst2 with 53% sequence identity. A BLAST search against the structural database identified several carboxyl esterases, mainly from extremophilic sources, as homologs with sequence identity at around 27–30% with the best sequence coverage at around 90%. These include enzymes AaEst2 and EstE1 used as models in MR, *P. calidifontis* esterase (PestE; 30% sequence identity; PDB 2YH2; Palm et al., 2011) and *A. fulgidus* carboxyl esterase (AFEST; 28%; PDB 1JJI; De Simone et al., 2001).

**FIGURE 3:**
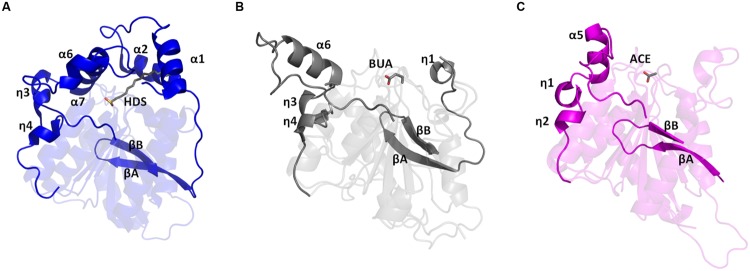
**Cartoon diagrams showing the protein structures of **(A)** AaEst2 (blue), **(B)** TtEst2 (gray) and **(C)** LpEst (magenta) in the same orientation.** The regions which form the ‘cap’ domain in AaEst2 and restrict the carboxyl and alcohol binding sites in TtEst2 and LpEst are highlighted and the secondary structure elements are labeled. The carboxyl reaction products in TtEst2 and LpEst and the tetrahedral reaction intermediate analog in AaEst2 are shown as stick models.

When compared to these four proteins, TtEst2 is lacking at least 30 amino acids at the N-terminus (**Figure 2A**). Within the α/β-hydrolase family 3 the ‘cap’ domain is formed by the two N-terminal α-helices and the insertion between βF and βG which contains two helices which shield the active site cavity (**Figure 3A**). In TtEst2 there is a minimal ‘cap’ domain, due to the absence of the N-terminal helices. Also, the helical insertion containing α6 and η3–η4 between βF and βG in TtEst2 is shorter than in the enzymes listed above and adopts a different conformation, pointing away from the active site region (**Figure 3B**). This insertion is likely to be a key factor in determining the substrate specificity as it defines the far end of the alcohol pocket. The loop region consisting of amino acid residues 172–176 within this insertion is poorly ordered in all of the TtEst2 structures suggesting significant flexibility within this region. These structural features result in TtEst2 having a very open active site pocket which is exposed to the solvent. The active site residues are located in an open shallow groove of the core domain.

On the other edge of the β-sheet a short loop connects α5 and βF in AaEst2. This loop is significantly extended in TtEst2 with a sequence insertion of seven residues and at the expense of a shorter α5 helix. This loop shields the α3 helix from solvent and is poorly ordered in the region of amino acid residues 144–149.

A DALI (Holm and Rosenström, 2010) search against TtEst2 revealed the closest structurally related carboxyl esterase to be Cest-2923 from *L. plantarum* (LpEst; PDB 4C01; Benavente et al., 2013) despite having only 22% sequence similarity. As with TtEst2, LpEst also lacks the majority of the ‘cap’ domain and LpEst is 15 residues shorter than TtEst2 at the N-terminus. LpEst has an even shorter insertion between βF and βG than TtEst2 which folds differently, pointing further toward the active site cavity (**Figure 3C**).

#### Quaternary Structure

Structural homologs of TtEst2 have different oligomeric states. While TtEst2 and AaEst2 structures are monomers in the crystal, PestE and EstE1 are tetramers and AFEST forms a stable octamer, with the same basic dimeric unit contributing to octamers and tetramers. The oligomeric structure does not seem to be important for catalysis, as the active site cavities appear to be distant from the monomer interfaces and the adjacent subunits do not contribute residues to the active sites. The TtEst2 enzyme would be unable to form such a basic dimer due to a potential steric clash of α6 and the loop 168–177 with their symmetry mates in such a dimer.

The LpEst forms a dimer which is very different from that of EstE1 with an adjacent subunit contributing to the active site cavity. TtEst2 would be unable to form such a dimer due to a clash of its N-terminus with a symmetry related molecule.

#### Crystal Soaks

The much reduced ‘cap’ domain in TtEst2 and its activity toward variable length carboxylic esters prompted interest as to the mode of substrate binding in the enzyme active site.

In the previously reported structural characterization of the first esterase to be identified from *T. terrifontis*, TtEst, we have established that soaking of the TtEst esterase crystals with the carboxylic acids at physiological pH resulted in the ligand binding with the hydrophobic carbon ‘handle’ pointing away from the carboxyl pocket of the active site, toward the alcohol pocket (Sayer et al., 2015). This is in agreement with the orientation of the ethyl ‘handle’ of the propionate ligand in the structure of the *Pseudomonas fluorescens* cofactor free choloroperoxidase determined at neutral pH (Hofmann et al., 1998; PDB 1A8S).

However, we have established that soaking of the TtEst crystals with carboxylic acids at low pH resulted in binding of the ligand with the ‘carbon handle’ positioned in the carboxylic pocket of the active site (Sayer et al., 2015). We have attributed this difference in the mode of binding of the carboxylic ligands at different pHs to an absence of charge on the carboxylic acids at the low pH.

Therefore we have used soaks of the TtEst2 crystals at pH 4.5 to position the carboxylic ligands into the carboxyl pocket. Indeed, soaks with the propionate and butyrate ligands have allowed the elucidation of the structures of the complexes of TtEst2 with the two carboxylic acid products bound in the carboxyl pocket.

Crystal soaks with the substrate *p*NP valerate were conducted at pH 4.5 with the objective to define both the carboxyl and alcohol binding pockets within the active site of the TtEst2 enzyme. In the crystal structure of the complex clear electron density was observed for the valerate component of the soaked ligand, but no continuous density was observed for either the *p*NP component of the ligand or the *p*NP product. This crystal structure will henceforth be called the valerate complex.

#### Active Site

**FIGURE 4:**
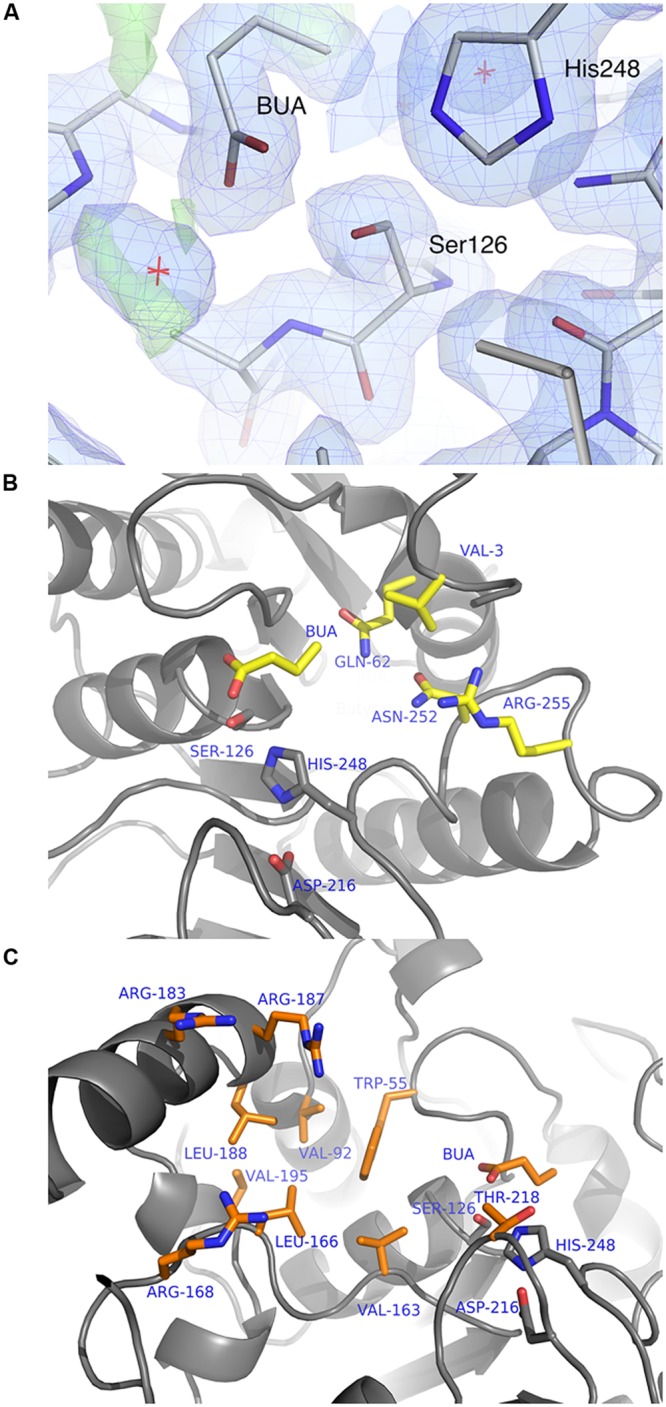
**(A)** The electron density maps showing the butyrate ligand (BUA) bound in the TtEst2 active site. The 2F_o_-F_c_ (blue) is contoured at 1.2 σ and the F_o_-F_c_ map is contoured at 3.5 σ (green) and -3.2 σ (red). The ligand and amino acid residues are shown as stick models. The catalytic Ser126 and His248 are annotated. **(B)** Structural features of the TtEst2 carboxyl binding site responsible for the binding of the substrate acyl group. The protein backbone is shown as a cartoon, the butyrate molecule (BUA) and the side chains of the carboxyl pocket amino acid residues are shown as stick models with carbon atoms in yellow and active site triad residues are shown with carbon atoms in gray. **(C)** Structural features of the TtEst2 alcohol binding site. The butyrate ligand (BUA) and the side chains of the amino acid residues lining the active groove are shown as stick models with carbons in orange, the active site catalytic residues in gray and the protein backbone is shown as a cartoon.

The crystals were soaked in a cryo-protectant solution containing propionate, butyrate or *p*NP-valerate at pH 4.5. The resulting structures revealed density for the bound ligands and in the *p*NP-valerate case, electron density was observed for the valerate part of the ligand. All three carboxyl ligands have similar modes of binding, although the butyrate molecule appears to be most clearly defined (**Figure 4A**). The direction of the substrate carbon handle is consistent with that observed in the complexes of TtEst (Sayer et al., 2015). The carboxyl group of these three reaction products is bound with the carboxyl carbon atom in close proximity to the catalytic serine (Ser126) while one of carboxyl oxygens is hydrogen bonded to the backbone amides of Gly53, Gly54, and Ala127 which form the oxyanion hole. This corresponds to the expected position that the carboxyl group of the ester substrate would occupy during the course of the reaction. In most other families of α/β-hydrolases the oxyanion hole is formed by two main chain nitrogens. It also requires two consecutive glycine residues in an extended conformation to form such an oxyanion hole, which may be beneficial for the hydrolysis. In the α/β-hydrolase family 3, Gly52, and Gly53 occupy positions 2 and 3 in the sequence and the structurally conserved GGG(aromatic residue) motif (**Figure 2A**). Such a three residue oxyanion hole was first reported for AaEst2 (De Simone et al., 2000) and appears to be a common feature within this family.

The carbon atoms of bound ligands thus map the carboxyl binding pocket of the TtEst2 active site. This site is defined as a shallow groove which is open to the solvent. It is restricted by the N-terminal 3_10_-helix, the loop between βC and α1 which contains the 3_10_-helix 61–64 and the loop 251–256 between βH and α8. This carboxyl binding pocket is lined by the aliphatic side chain of Val3 and the polar/charged side chains of residues Glu62, Asn252, and Arg255 (**Figure 4B**). The side chain of Val3 limits the length of the bound carboxyl part of the ester substrate and the polar character of the open groove favors activity toward shorter carboxyl chain *p*NP-esters as it affects binding of the longer hydrophobic tails. Introduction of aliphatic/aromatic residues into this groove may prove favorable for hydrolysis of longer chain substrates.

The alcohol part of the ester substrate of TtEst2 would bind to a shallow groove on the surface of the protein that is open to solvent. The helical region between βF and βG which forms the far wall of this groove is approximately 14 Å away from the catalytic serine residue. The hydrophobic side chains of Trp55, Val92, Leu188, Leu166, Val163 line the bottom of this groove with positively charged Arg168, Arg183, and Arg187 located at the far edge of groove (**Figure 4C**). Ester substrates with short hydrophobic alcohol components are likely to bind at the bottom of this pocket, while more extended substrates with a distal carboxyl group would bind to one of the arginine residues.

Although the TtEst2 active site is open there is no evidence for promiscuity within the α/β-hydrolase family 3 which has been reported for the *P. aeruginosa* lysophospholipase TesA which also has an open active site (Kovačić et al., 2013).

#### Carboxyl Binding Site Comparison

There are two reported structures of α/β-hydrolase family 3 enzymes, LpEst and AaEst2, which have ligands bound in the carboxyl binding pocket of the active site.

**FIGURE 5:**
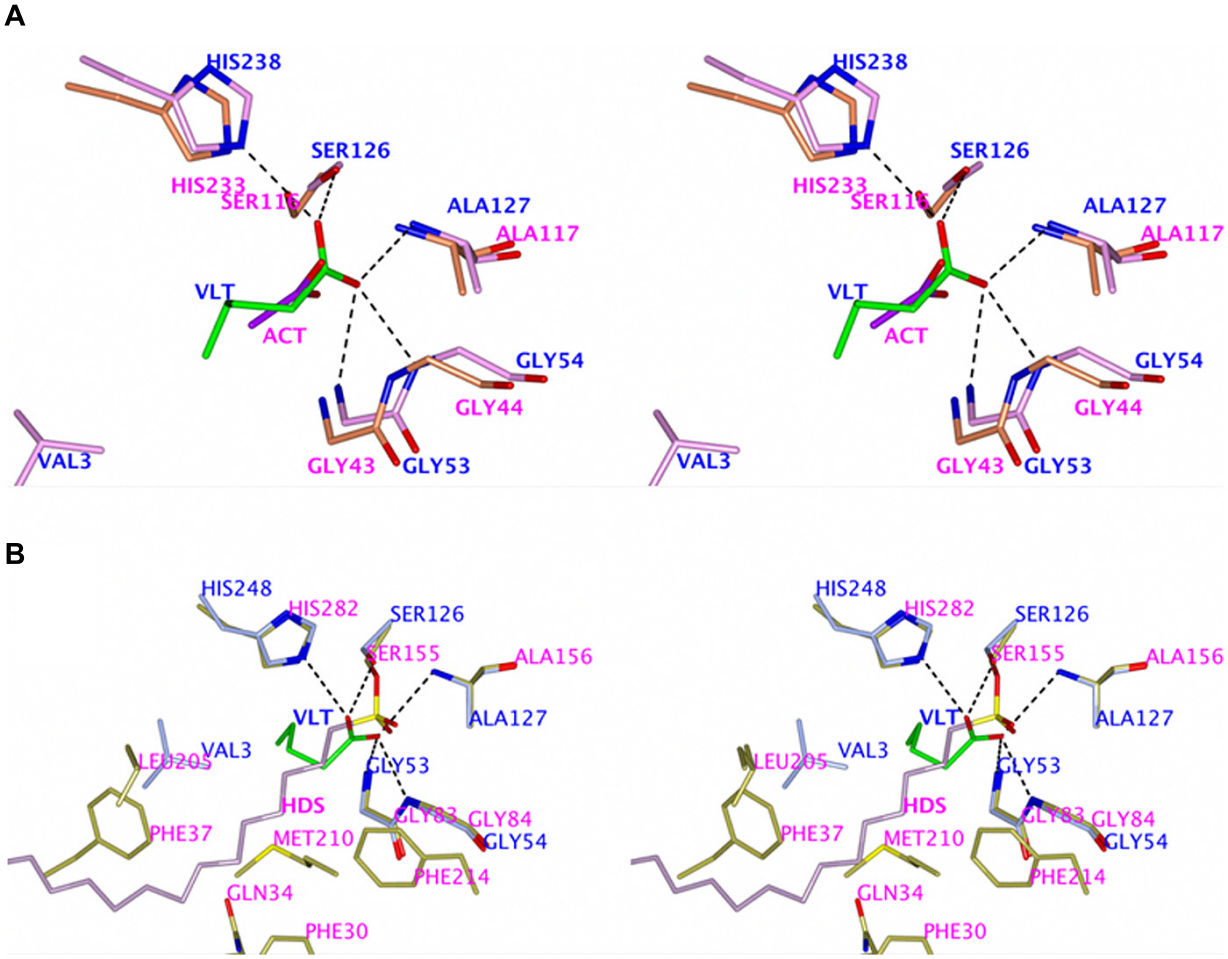
**(A)** Stereo diagram showing the superimposition of the carboxyl binding pockets of the acetate (ACT) complex of the LpEst and the valerate complex of TtEst2 (VLT in green). The key residue side chains are shown as sticks models. Hydrogen bonding for the ligand in TtEst2 showing the three main chain nitrogen oxyanion hole is shown as black dashed lines. **Figures 5** and **6** were prepared using CCP4mg (McNicholas et al., 2011). **(B)** Stereo diagram showing the superimposition of the 1-hexadecanesulfonyl (HDS) complex of the AaEst2 and the valerate complex of TtEst2 (VLT in green). The key residue side chains are shown as sticks and the hydrogen bonding for the valerate substrate in TtEst2 as black dashed lines.

A comparison of the valerate binding in TtEst2 and the acetate ligand binding in LpEst (PDB 4C01) shows a similar positioning of the carbon handle (**Figure 5A**) which reveals several differences in the carboxyl binding pocket. The N-terminus in the TtEst2 folds into a 3_10_-helix which positions the side the chain of Val3 in a restrictive position in the active site cavity. The LpEst is several residues shorter at the N-terminus and is lacking this feature. Instead a loop between β2 and β3 comes from an adjacent subunit in the LpEst dimer to limit the carboxyl binding pocket in this enzyme. The carboxyl pocket in LpEst is also limited by a longer loop region between βH and α8 compared to TtEst2. Both LpEst and TtEst2 have carboxyl pockets of comparable size lined with polar/charged residues which favor shorter carboxyl acid esters. Indeed, both enzymes have preference to *p*NP-acetate (Benavente et al., 2013) and decreasing activity toward larger substrates.

**FIGURE 6:**
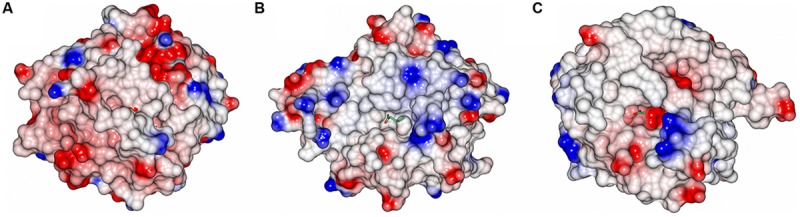
**The electrostatic potential surface of the AaEst2 HDS complex **(A)**, TtEst2 valerate complex **(B)** and LpEst acetate complex **(C)** showing the overall large difference between the enzymes.** The active sites of TtEst2 and LpEst are shallow grooves open to solvent while the ‘cap’ domain in AaEst2 covers the inhibitor. The positive charge is shown in blue and the negative charge is shown in red. The ligand molecules were not used in the surface calculation and are shown as sticks.

A comparison of the TtEst2 carboxyl complexes to the AaEst2 irreversible covalent complex with 1-hexadecanesulfonyl (PDB 1QZ3; De Simone et al., 2004) shows that the long hydrophobic carbon tail mimicking the carboxyl group of its substrate has the same direction as the carbon handles in TtEst2 and LpEst, however, it is much longer and goes into the ‘cap’ domain of AaEst2 which is absent in TtEst2 and LpEst (**Figure 5B**). Surface representation of AaEst2 with its bound ligand (**Figure 6A**) shows that the substrate is protected from solvent by the ‘cap’ domain, while in TtEst2 and LpEst the carboxyl pockets are open to solvent (**Figures 6B,C**). The very different carboxyl pocket of AaEst2 which extends into the ‘cap’ domain appears to define its preference to longer carboxyl substrates of C4–C6 length (De Simone et al., 2004).

#### Alcohol Binding Site Comparison

The helical insertion between βF and βG has different lengths and adopts different conformations in the TtEst2, LpEst, and AaEst2 enzymes, the latter representing α/β-hydrolase class 3 esterases which have a full ‘cap’ domain.

In the AaEst2 the active site is covered by a ‘cap’ domain where the alcohol pocket is in a channel deep inside the protein and can be mapped by a covalent adduct of a HEPES molecule that is bound to the active site serine to form the tetrahedral intermediate between the domains (De Simone et al., 2001). The insertion after βF forms part of the AaEst2 ‘cap’ domain which contributes to both the alcohol pocket and the carboxyl binding pocket (**Figure 3B**). The side chains of residues lining the pocket define the substrate specificity for enzymes with a ‘cap’ domain.

The LpEst and TtEst2 have no N-terminal α-helices to form part of the ‘cap’ domain. The active sites of these two enzymes are solvent exposed grooves, however, these two enzymes have quite different organization of their active sites.

The region following βF is six residues shorter in TtEst2 compared to AaEst2 and points away from the active site (**Figure 3B**). In addition the position of α7 in AaEst2 which is not present in TtEst2 gives a much more defined distinctive alcohol pocket. This helix projects Phe214 and Arg215 into the active site of AaEst2 which is an area that is open to the solvent in TtEst2.

In LpEst (**Figure 3C**) the insertion after βF is eight residues shorter than in TtEst2, but folds back toward the catalytic serine limiting the size of the alcohol pocket to about half of that in TtEst2. It is lined by hydrophobic side chains with no charged residues to bind any polar groups of the alcohol part of the substrate (**Figure 6C**). This poses a question of whether the conformation of this helical region between βF and βG is an artifact of crystallization and if the insertion can adopt a different conformation pointing toward the active site. It would appear unlikely as the residues of this region in TtEst2 form hydrogen bonds with other structural elements and there are no obvious hinges in this region that would allow movement.

#### TtEst2 Thermostability

The TtEst2 is an exported protein, as its sequence contains a leader peptide. It has a reasonable degree of thermostability as expected for its role in the moderate thermophile, *T. terrifontis.* It appears to be less thermostable than its homologs with a ‘cap’ domain, EstE1 (Rhee et al., 2005) and AaEst2 (Del Vecchio et al., 2002) or the TtEst from the same organism (Sayer et al., 2015). The TtEst2 is a monomer, while oligomerisation is one of the mechanisms of adaptation to elevated temperatures (Singleton et al., 1999; Littlechild, 2011). Dimerization was reported to contribute to the EstE1 thermostability (Byun et al., 2007). There are three regions in the TtEst2 structure that are not well ordered and are likely to reduce its thermostability. These are the flexible N-terminus and the loop residues 143–152 and 170–180. The comparable regions are well ordered in the EstE1 and AaEst2 crystal structures.

## Conclusion

The TtEst2 represents a novel thermophilic carboxyl esterase that differs significantly from an earlier described carboxyl esterase from the same organism which has been identified as the first thermophilic Planctomycetes species. The two carboxyl esterase enzymes are thought to play different roles in the *Thermogutta* cell since TtEst2 is exported into the periplasm due to its signal peptide and the earlier studied TtEst is located in the cytoplasm. The TtEst2 enzyme reveals an open substrate binding site, which is solvent accessible due to the absence of the usual ‘cap’ domain which is present in TtEst. Comparison of the TtEst2 with related structures has provided explanation of its substrate specificity. This information is important for the potential applications of thermophilic esterases for industrial processes.

### Accession Codes

The GenBank sequence accession number for the *T. terrifontis* carboxyl esterase *TtEst2* is KT724966.

The protein structures for the *T. terrifontis* carboxyl esterase and their complexes have been deposited in the Protein Data Bank with codes: 5AO9 (native), 5AOA (propionate bound), 5AOB (butyrate bound), 5AOC (valerate bound).

## Author Contributions

CS carried out biochemical and structural characterisation of the enzyme. CI directed work at MicroDish. MI was involved in the structural determination of the enzyme. ZS cloned and over-expressed the enzyme. JL oversaw the project. CS, MI, and JL contributed to the writing of the manuscript.

## Conflict of Interest Statement

The authors declare that the research was conducted in the absence of any commercial or financial relationships that could be construed as a potential conflict of interest.

## Abbreviations

AaEst2: *Alicyclobacillus acidocaldarius* esterase
AEXII: *Penicillium purpurogenum* acetylxylan esterase
AFEST: *Archaeoglobus fulgidus* carboxylesterase
EstE1: a metagenomic thermophilic carboxylesterase
LpEst: carboxylesterase Cest-2923 from *Lactobacillus plantarum*
PestE: esterase from *Pyrobaculum calidifontis*
TtEst: esterase from *Thermogutta terrifontis*
TtEst2: esterase2 from *Thermogutta terrifontis*
